# Integration of Postbiotics into Adult Diapers: *In Vitro* Evaluation of Biocompatibility and Effect on Skin Microbiota

**DOI:** 10.3390/life15111652

**Published:** 2025-10-23

**Authors:** Oznur Ozlem Ibrahimoglu, Leyla Tarhan Celebi, Dilan Ece Dikbiyik, Halise Betul Gokce, Bekir Cakici, Zafer Türkoğlu, Ayse Nilhan Atsu, Ismail Aslan

**Affiliations:** 1 Hayat Kimya R&D Center, Kocaeli 41275, Türkiye; ozlem.ibrahimoglu@hayat.com.tr (O.O.I.); dilanece.dikbiyik@hayat.com.tr (D.E.D.); 2 SFA R&D Co., Ltd., Teknopark İstanbul, İstanbul 34890, Türkiye; leyla.tarhan@sfaarge.com (L.T.C.); bekir.cakici@sfaarge.com (B.C.); 3 ATABIO Technologies Co., Ltd., Teknopol İstanbul, İstanbul 34903, Türkiye; 4 Department of Pharmaceutical Technology, Faculty of Pharmacy, Afyonkarahisar Health Sciences University, Afyonkarahisar 03030, Türkiye; betul.aslan@afsu.edu.tr; 5 Vocational School, İstanbul Kent University, İstanbul 34406, Türkiye; aysenilhan.atsu@kent.edu.tr; 6 Department of Dermatology, Başakşehir Çam & Sakura Hospital, İstanbul 34480, Türkiye; zafer.turkoglu@sbu.edu.tr; 7 Hamidiye Faculty of Pharmacy, Department of Pharmaceutical Technology, University of Health Sciences, İstanbul 34668, Türkiye; 8 Faculty of Pharmacy, İstanbul Kent University, İstanbul 34406, Türkiye

**Keywords:** postbiotics, nonwoven fabrics, skin microbiota, adult diapers, cytotoxicity, lactic acid quantification, microbiota friendly, ATA-coded postbiotics

## Abstract

Postbiotics are bioactive microbial metabolites recognized for their potential to support skin health and balance the microbiota. In this study, nonwoven fabrics and adult diaper prototypes, with and without postbiotic incorporation, were evaluated for their effects on skin microbiota, epidermal integrity, and cytotoxicity. *In vitro* assays using reconstructed human epidermis and keratinocyte cell lines demonstrated that postbiotic-containing samples maintained high tissue and cell viability. Microbiota diversity analyses confirmed that postbiotic formulations maintained a favorable ratio of *Staphylococcus epidermidis* to *Staphylococcus aureus*. Collectively, these findings indicate that ATA-coded postbiotic-embedded nonwoven and adult diaper prototypes are skin microbiota-friendly, safe for epidermal contact, and stable in their bioactive compound content. These results underscore the potential of postbiotics as functional agents in personal hygiene products to promote skin health.

## 1. Introduction

Skin is inhabited by a rich and adaptable community of microorganisms—bacteria, fungi, viruses, and archaea—that occupy both its surface and appendages. The composition of this microbial ecosystem varies markedly between anatomical regions, such as dry, moist, and sebaceous sites, each of which sustains distinct bacterial communities. Prominent bacterial genera such as *Staphylococcus* and *Cutibacterium* support skin health by controlling pathogen expansion and enhancing barrier function. Moreover, metabolites produced by the skin microbiota modulate immune responses and sustain barrier homeostasis, thereby safeguarding against inflammation and infection [1,2].

An increasing number of studies demonstrate that the skin microbiota contributes significantly to wound healing, immune homeostasis, and skin well-being, indicating that modulation of microbial populations could lead to novel treatment strategies [1].

The equilibrium between *Staphylococcus aureus* and *Staphylococcus epidermidis* on the skin represents a dynamic balance that plays a crucial role in maintaining cutaneous health. *S. epidermidis* is a predominant commensal species that strengthens the skin barrier and inhibits the colonization and virulence of pathogens like *S. aureus* by competing for resources and releasing antimicrobial peptides [3]. Conversely, *S. aureus* is an opportunistic pathogen that can trigger a range of infections, and excessive colonization by this species is closely linked to skin diseases such as atopic dermatitis [4].

Recent studies have highlighted that the *S. aureus–S. epidermidis* equilibrium can be disrupted by antibiotic use, impaired immunity, or barrier disruption, leading to increased *S. aureus* colonization and heightened susceptibility to infection [4,5].

Understanding the molecular mechanisms and ecological dynamics that preserve this balance is crucial, since directed manipulation of the skin microbiota may offer novel approaches to prevent and manage *S. aureus* related infections [3,5].

The human skin microbiota is defined as the diverse and dynamic community of microorganisms that contribute to cutaneous homeostasis, immune regulation, and defence against pathogenic invasion. Disturbances in this ecosystem have been implicated in the etiology of acne, atopic dermatitis, and irritant dermatitis, particularly in vulnerable populations such as adult diaper users. In order to address these challenges, interest has shifted from conventional antimicrobials to microbiota-supportive approaches. In this context, postbiotics—non-viable microbial cells, fractions, or metabolites—have gained increased recognition for their safety, stability, and beneficial bioactivities in comparison with live probiotics [6,7,8,9].

Postbiotics are defined as non-viable microbial products or metabolic byproducts that exert physiological benefits to the host [10]. Their documented properties include anti-inflammatory, antioxidant, immunomodulatory, antimicrobial, and anticancer effects, many of which are directly relevant to skin integrity and wound repair [11,12,13]. Several *in vitro* and *in vivo* studies have demonstrated the potential of postbiotic formulations to accelerate fibroblast proliferation, enhance collagen synthesis, suppress pro-inflammatory cytokines (e.g., IL-6, TNF-α), and promote epithelial regeneration in chronic wounds [14,15,16,17].

Postbiotics, such as lactic acid, short-chain fatty acids, peptides, and cell-wall fragments, have demonstrated immunomodulatory, anti-inflammatory, and barrier-reinforcing effects [18,19,20]. A plethora of clinical and preclinical studies have reported improvements in hydration, reduced transepidermal water loss, and enhanced microbial balance following topical application of postbiotic formulations. These findings suggest their potential in skincare, wound healing, and dermatological applications [21,22,23].

A number of recent investigations have confirmed the validity of these outcomes in a variety of product contexts. For instance, Aslan and Tarhan Çelebi demonstrated that a postbiotic-enriched antiperspirant suppressed malodor-associated microorganisms while preserving axillary microbial diversity and keratinocyte viability [24]. In a complementary approach, Gökçe and Aslan developed a liposomal gel delivery system for postbiotics, showing improved stability, controlled release, and preserved antimicrobial activity—highlighting formulation strategies that enhance functional reliability [25]. In addition, Tarhan Çelebi and colleagues reported that postbiotics derived from fermented dairy matrices exhibited a protective effect on normal fibroblasts while selectively reducing the viability of colon cancer cells, thereby illustrating their versatile bioactivity profile [26].

These observations are consistent with global findings: topical lotions containing *Lactobacillus plantarum* lysates have been shown to reduce acne lesions while strengthening barrier integrity, and yeast-extract-based postbiotics have been demonstrated to improve both barrier function and microbial richness in comparison to conventional moisturisers [27,28]. Mechanistic studies further demonstrate that postbiotics accelerate wound closure in *ex vivo* human skin and enhance tight-junction protein expression, reinforcing epithelial resilience [29,30,31].

Notwithstanding these encouraging advances, the systematic incorporation of postbiotics into nonwoven hygiene textiles remains largely unexplored. Adult diaper topsheets and nonwoven substrates are in prolonged contact with the skin under moist and occlusive conditions, which can result in irritation, microbial imbalance, and barrier disruption [32]. The incorporation of postbiotics into these textile matrices could be a pioneering approach to alleviate diaper-associated dermatitis while fostering microbiota homeostasis.

The present study evaluates the integration of postbiotics into nonwoven fabrics and adult diaper prototypes. Specifically, the investigation encompasses three key areas: (i) the incorporation and stability of postbiotics, utilising gravimetric oil pick up (OPU) determination and HPLC-UV quantification of lactic acid; (ii) the assessment of biological safety, employing keratinocyte toxicity assays (ISO 10993-5) and reconstructed epidermis irritation tests (OECD 439); and (iii) the analysis of microbiota interactions, with a particular focus on the *S. epidermidis*/*S. aureus* equilibrium [33,34]. The objective of the present study is to establish a foundation for the development of microbiota-friendly, skin-safe hygiene products.

## 2. Materials and Methods

### 2.1. Test Materials

Postbiotic formulations from SFA R&D (İstanbul, Türkiye) were mixed into an aqueous solution of wetting agent. The postbiotic solution was prepared at a concentration of 5% (*w*/*v*) in the wetting agent.

The composition of the tested postbiotics is summarized below:

Enumeration of Total Viable Bacteria (after tindalization): <10 cfu/g;

Lactobacillus paracasei*ATA-LTC-Lp041103: 1.00 × 10^10^ cfu/g;

Lactobacillus plantarum*ATA-LPC98052: 1.00 × 10^10^ cfu/g;

Bifidobacterium longum* ATA-BSP1908: 1.10 × 10^10^ cfu/g;

Streptococcus thermophilus* ATA-LTC-Ld120812: 1.00 × 10^10^ cfu/g.

The complete composition of the tested postbiotics is provided for clarity.

*Total viable count values refer to measurements before tindalization.

The species frozen in cryotubes were first brought to room temperature. Lactobacillus and Streptococcus strains were streaked onto MRS agar, while Bifidobacterium strains were streaked onto BHI agar and incubated at 37 °C for 24 h. After incubation, colonies were collected from the agar and transferred into liquid media (MRS or BHI) and homogenized to a McFarland standard of 1. The resulting homogenate (10 mL) was inoculated into a fermenter, which was operated at 37 °C, 150 RPM for 24 h. At the end of fermentation, the entire content was autoclaved at 121 °C for 30 min. The postbiotic-containing solution was then filtered through a 0.45 µm membrane before use.

Nonwoven spunbond Evony^®^ topsheets were supplied and produced from Hayat Kimya A.Ş. R&D (Kocaeli, Turkiye). Postbiotic solution was evenly applied to the spunbond nonwoven topsheet using a kiss-roll coater. The topsheet was air-dried at 85–90 °C. The dried nonwoven material (Evony^®^) was then incorporated into adult diapers on Hayat Kimya A.Ş. R&D’s production machines for further testing, stored under sterile, sealed conditions.

All nonwoven fabrics and adult diaper prototypes, with and without postbiotic incorporation, were supplied by Hayat Kimya A.Ş. R&D laboratories. All ATA-coded prototypes were tested in parallel to allow paired evaluation of postbiotic and non-postbiotic versions.

The tested groups were

Adult diaper prototypes (controls): D100, D200, D300 (nonwoven references without postbiotics).

Adult diaper prototypes (postbiotic-embedded): D100-P, D200-P, D300-P (prototypes containing postbiotics).

Nonwoven topsheet fabrics: 14 g/m^2^ nonwoven structures, prepared with or without postbiotic incorporation.

Solutions and sprays: concentrated formulations containing a wetting agent and either 2.0 or 2.2 mL of postbiotic suspension.

**Figure 1:**
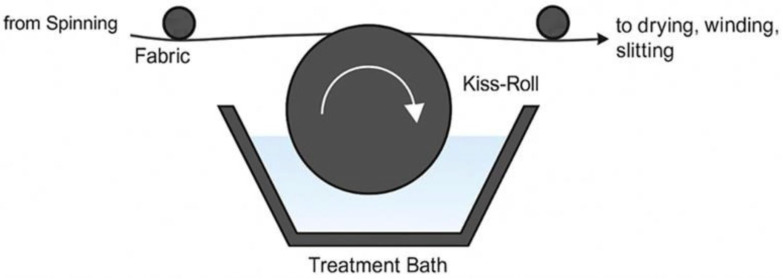
Graphical representation of the kiss roll process for nonwoven fabrics, where an immersed roll carries liquid from the reservoir and transfers it to the fabric through controlled contact. The arrow indicates the flow direction of the nonwoven fabric.

Application of Postbiotic Formulations onto Nonwoven Topsheets: A schematic of the kiss-roll coating process for nonwoven fabrics is shown in Figure 1. In this process, an immersed roll picks up a liquid from a reservoir and transfers it to the fabric via controlled contact, allowing uniform application.

Nonwoven spunbond topsheets were supplied and produced by Hayat Kimya (Kocaeli, Turkiye). Postbiotic formulations obtained from SFA (İstanbul, Turkiye) were incorporated into an aqueous solution designed to enhance fabric wettability. The mixture was applied to the spunbond nonwoven topsheets at a controlled application level, using a kiss-roll coater to ensure consistent distribution across the material surface.

Following coating, the topsheets were air-dried until reaching constant weight. The dried nonwoven materials were subsequently integrated into adult diaper assemblies using Hayat Kimya’s production lines. Finished samples were stored under sterile, sealed conditions until further evaluation.

### 2.2. Postbiotic Characterization and Quantification

#### 2.2.1. Gravimetric Determination of Oil Pick-Up Capacity in Posbiotic-Treated Nonwoven Fabrics

The gravimetric method was used to quantify OPU within the samples. Approximately 4–5 g of each nonwoven specimen was extracted with 100 mL absolute ethanol using a filtration setup. The solvent was evaporated under controlled heating, and residues were dried to constant weight in an oven. The percentage of OPU was calculated based on the weight difference. Each determination was performed in six replicates per analyst.

#### 2.2.2. Quantitative Determination of Lactic Acid in Postbiotic-Containing Nonwoven Textiles by HPLC-UV

Lactic acid, as a marker postbiotic metabolite, was quantified using a modified method adapted from ACM010 (Philippines FDA). A reversed-phase HPLC system with UV detection was used. Calibration curves were constructed at concentrations of 0.5–5 ppm (R^2^ ≥ 0.995). Nonwoven specimens were cut into equal areas (~630 cm^2^), extracted with 50 mL phosphate buffer, sonicated for 20 min, filtered, and injected into the HPLC column. Results were expressed as % lactic acid. All analyses were performed in six replicates per analyst, and data were statistically evaluated using Grubbs’ test (to detect potential outliers in the dataset) and ANOVA (analysis of variance) tests.

### 2.3. In Vitro Cytotoxicity Assays

#### 2.3.1. Keratinocyte Cytotoxicity Test (ISO 10993-5)

Human keratinocyte cell line (HEKA 500K, SN 34120) was cultured under standard conditions (37 ± 1 °C, 5 ± 0.5% CO_2_, 75 ± 5% RH). Cells were exposed to sample extracts or by direct contact for 72 h. The same extracts were consistently used throughout the study. Extracts were prepared by cutting the test materials into pieces corresponding to 3 cm^2^/mL, immersing them in DMEM cell culture medium supplemented with bovine serum, and incubating at 37 °C for 24 h in an orbital shaker incubator. After incubation, the mixtures were vortexed, and the liquid phase (extract) was collected for use in the experiments. For each well of the 24-well plate, 2 mL of extract was applied. A 10% (*w*/*v*) sodium dodecyl sulfate (SDS) solution was used as the positive control. Control samples were prepared in the same manner as the test samples: cut into 3 cm^2^/mL pieces, immersed in DMEM medium supplemented with bovine serum, incubated at 37 °C for 24 h in an orbital shaker incubator, vortexed, and the liquid phase was collected for use. All treatments were performed in triplicate [33].

The MTT assay was performed to assess cell metabolic activity, with absorbance measured at 570 nm. Controls included Dulbecco’s PBS (negative), polyethylene CRM (non-cytotoxic), and sodium dodecyl sulfate 2.3% (positive). Viability ≥ 70% was considered non-cytotoxic [35].

#### 2.3.2. Reconstructed Human Epidermis Irritation Test (OECD 439)

Reconstructed epidermal tissues (EPISKIN EPI200, SN 36169; It is created by growing normal human keratinocytes (skin cells) on a collagen matrix at the air-liquid interface) were exposed to 30 µL of test samples for 1 h, followed by rinsing with DPBS and incubation for 24 h. Viability was quantified by MTT reduction assay and compared to negative controls. Tissue viability ≥ 50% classified samples as “non-irritant.” Positive control (SDS) resulted in viability ≤ 10% [34].

### 2.4. Microbiota Interaction Studies

#### 2.4.1. Selection of Skin Microorganisms

Representative skin microbiota strains were selected, including: *Lactobacillus crispatus ATA-LTC 240522*, *Staphylococcus epidermidis ATCC 12228*, *Staphylococcus aureus ATCC 6538*, *Staphylococcus capitis ATCC 35661*, *Cutibacterium acnes ATA-LPC 0204221*, *Streptococcus pyogenes ATA-TCSP 210911* and *Candida albicans ATCC 10231*. They were used in this study as they are commonly associated with the human skin microbiome. Inocula were standardized to ~3.5 × 10^2^–5.0 × 10^2^ CFU/mL depending on test series.

#### 2.4.2. Assessment of Microbial Load of Postbiotic-Containing Nonwoven 

Microbiological suitability tests were performed to prove that the patient’s diapers are microbiologically clean. All samples passed microbiological safety limits (absence of *E. coli*, *P. aeruginosa*, *S. aureus*, *C. albicans*) [36,37,38,39,40,41,42]. To assess the microbiological suitability of the samples, a direct plating assay was performed to confirm that they did not carry any microbial load. For each sample, a 1 cm^2^ piece was placed onto an empty Petri dish, and molten general growth medium (e.g., TSA), cooled to 45–50 °C, was poured over it. The plates were incubated at 37 °C for 72 h. The same procedure was applied using PDA to assess molds and yeasts, with plates incubated at 22 °C for 5–7 days.

#### 2.4.3. Inoculation and Exposure Conditions

Test materials (adult diaper topsheets and solutions) were inoculated with microorganisms for 1–4 h contact times at 37 ± 1 °C. In immobile patients, diaper changes are performed every four hours. Therefore, a contact time of four hours was used. Experiments were conducted under ISO 17025-accredited microbiology laboratory conditions (biosafety ISO class 7–8) [34].

#### 2.4.4. Microbial Balance and Microbial Diversity Assessment

The balance between S. epidermidis and S. aureus was specifically monitored to assess skin microbiota–friendly effects, as described before [43]. Log10 CFU/mL values were used to calculate microbial diversity indices. Artificial skin microbiota—Skin microbiota simulated species applied on human epidermal keratinocytes: *Lactobacillus crispatus ATA-LTC 240522*, *Staphylococcus epidermidis ATCC 12228*, *Staphylococcus aureus ATCC 6538*, *Staphylococcus capitis ATCC 35661*, *Cutibacterium acnes ATA-LPC 0204221*, *Streptococcus pyogenes ATA TCSP 210911*, and *Candida albicans ATCC 10231* [44,45].

### 2.5. Statistical Analysis

Data from OPU and lactic acid determinations, cytotoxicity assays, and microbiota tests were expressed as mean ± standard deviation (SD). Normality was confirmed, and outlier analysis was conducted with Grubbs’ test (α = 0.05). Variability and reproducibility were assessed by ANOVA. Results with *p* > 0.05 indicated no significant difference between analysts or test replicates.

## 3. Results

### 3.1. Gravimetric Determination of Oil Pick-Up Capacity in Posbiotic-Treated Nonwoven Fabrics 

Mean OPU content of postbiotic in Nonwoven samples was found between 0.64 and 0.68%. The measurement results obtained by different analysts are very close to each other and do not contain any outliers. This confirms that the analytical method used meets quality control standards. Studies have shown that the average OPU is between 0.64–0.68%. Reproducibility between analysts and replicates: RSD < 1% and ANOVA showing no significant inter-analyst variation (*p* > 0.05). The reliability, reproducibility, and precision of the method used to determine the OPU content in non-woven fabrics are presented in Table A1.

### 3.2. Quantitative Determination of Lactic Acid in Postbiotic-Containing Nonwoven Textiles by HPLC-UV 

Lactic acid was used as a marker for postbiotic quantification. The reliability, repeatability, and precision of the method used to determine the lactic acid content in non-woven fabric are presented in Table A2. The measurement results obtained by different analysts are very close to each other and do not contain any outliers. This confirms that the analytical method used meets quality control standards. Studies have shown that the average lactic acid concentration is between 0.0041% and 0.0045%.

The calibration curve for lactic acid analysis showed excellent linearity (R^2^ = 0.9991), confirming the method’s reliability and accuracy within the tested concentration range.

### 3.3. In Vitro Cytotoxicity and Tissue Viability

#### 3.3.1. Keratinocyte Cytotoxicity (ISO 10993-5)

**Figure 2:**
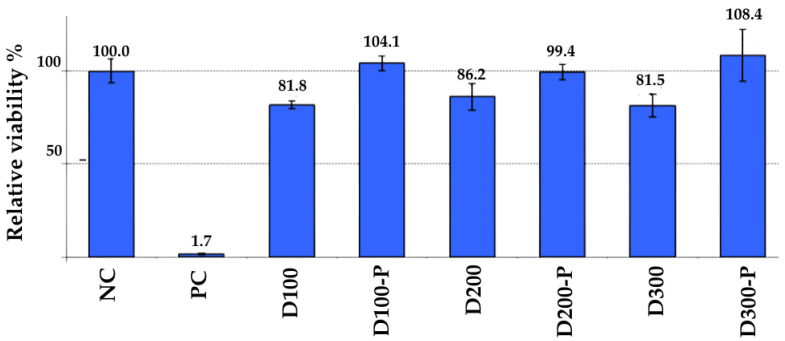
Relative viability of different groups. NC (Negative Control): This group is a reference that shows how healthy cells are in a non-toxic environment. Its value is set at 100.0%. This indicates that the cells have full viability under normal conditions. PC (Positive Control-SDS): This group shows how much cell viability decreases when a known cytotoxic (toxic to cells) substance is used. Its value is 1.7%. This proves that the test is working correctly and can reliably detect a cytotoxic effect. The samples with codes D100, D200, and D300 are materials that do not contain postbiotics, while samples with codes D100-P, D200-P, and D300-P contain postbiotics.

The main objective of this method is to determine whether the material being studied has a harmful effect on living cells. The test is performed *in vitro* in a laboratory environment, without the need for animal testing. The data obtained indicates whether the material has a cytotoxic effect on cells. If the material is found to reduce cell viability by more than 30% or cause significant morphological damage, it is considered cytotoxic. This means the expected cell viability should be >70%. In the conducted studies, it was observed that adult diapers containing postbiotics did not harm cell viability, with the living keratinocyte rate being >90%. In contrast, the viability was <90% in patient diapers without postbiotics. This is a promising development, suggesting that the addition of postbiotics may play a role in the cellular repair mechanism. The results are summarized in Figure 2.

#### 3.3.2. Epidermal Irritation Assay (OECD 439)

The OECD 439 Test uses 3D reconstructed human epidermis (RhE) models, which are produced in a laboratory and closely resemble real human epidermis [34]. These models are made of living keratinocyte cells and mimic the layered structure of the epidermis. If a substance reduces the cell viability of the skin model below a certain threshold (usually 50%), it is classified as an irritant. If cell viability remains above that threshold, the substance is considered a non-irritant. This study clearly shows that all non-woven fabric samples containing postbiotics are non-irritants, with tissue viability greater than 90%. These test results indicate that adult diapers containing postbiotics are safe and biocompatible in terms of their potential for skin irritation. According to results, all postbiotic samples were classified as “non-irritant”. Also, tissue viability ≥ 95% compared to negative controls.

**Figure 3:**
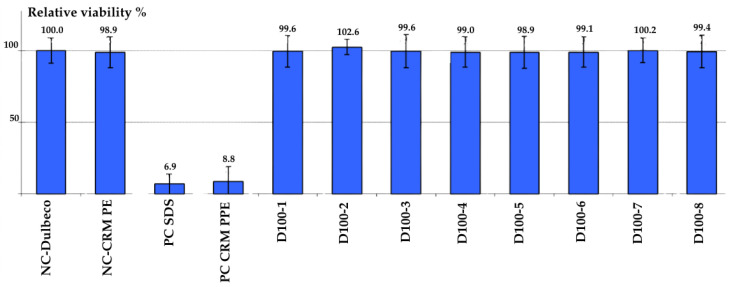
Evaluation of Cell Relative Viability in Weko Trial Samples with Postbiotic Addition.

Cell viability was evaluated for eight identical samples obtained from Weko-assisted trial production. Each sample was treated with a postbiotic compound to assess its effect on cellular viability. The results are summarized in Figure 3.

NC-Dulbecco: This is the negative control group, which shows the viability of cells in a normal, non-toxic environment. Its value is 100.0%. This indicates that the cells have full viability under normal conditions and serves as the baseline reference for the test. NC-CRM PE: This is a certified reference material used as a negative control (CRM, Certified Reference Material; PE, Polyethylene). Its value is 98.9%. This result confirms that polyethylene is a non-irritant and that the test method is working correctly. PC-SDS: As a positive control group, a known irritant substance, SDS (Sodium Dodecyl Sulfate), was used. Its value is 6.9%. Since it reduces cell viability well below the 50% threshold, it demonstrates that the test can reliably detect an irritating effect. PC-CRM PPE: This is another certified reference material used as a positive control (PPE, Polypropylene). Its value is 8.8%. This also shows an irritating effect, reinforcing the test’s accuracy. The graph includes a multi-replicate study of samples containing postbiotics. According to the OECD 439 test, a substance is classified as an irritant if it reduces cell viability below the 50% threshold. The multi-replicate study of postbiotic-containing samples was performed in the test. All of these samples-maintained cell viability between 99.6% and 106.2%. These values are well above the 50% threshold. One sample even showed a higher viability rate than the negative control. This graph clearly demonstrates that all non-woven fabric samples containing postbiotics are non-irritants. The control groups (Negative and Positive Controls) support the reliability and accuracy of the test. These test results show that the materials in question are safe and biocompatible in terms of skin irritation potential.

### 3.4. Effects on Skin Microbiota

#### 3.4.1. Assessment of Microbial Load of Postbiotic-Containing Nonwoven

Microbiological suitability tests were conducted to verify the microbiological cleanliness of patient diapers. All analyzed samples met the microbiological safety limits that indicates the absence of *Escherichia coli*, *Pseudomonas aeruginosa*, *Staphylococcus aureus*, and *Candida albicans*. The analyses were performed in accordance with ISO 21149:2017, ISO 22718:2015, ISO 22717:2015, ISO 16212:2017, ISO 18416:2015, ISO 21150:2015, and ISO 17516:2014 standards. All tested diaper samples complied with the microbiological quality requirements defined in ISO 17516:2014. The results are summarized in Table 1. No pathogenic microorganisms were detected.

#### 3.4.2. *S. epidermidis*/*S. aureus* Balance

**Figure 4:**
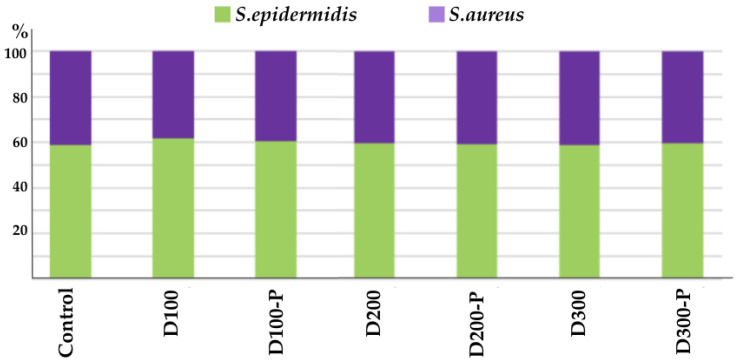
Graphical representation on *S. epidermidis/S. aureus* balance of different groups.

The balance analysis between *S. aureus* and *S. epidermidis* is important for evaluating a product’s compatibility with the skin microbiome. *Staphylococcus epidermidis* is a key part of the skin microbiome. It protects the skin against harmful microorganisms and supports the skin’s immune system. *Staphylococcus aureus* is normally found in small numbers on the skin, but when it overgrows, it can cause skin infections, acne, and conditions like atopic dermatitis. The purpose of the balance analysis is to see how a product (such as adult diapers) affects the delicate balance between these two bacteria. Ideally, a product should protect the beneficial *S. epidermidis* while inhibiting the proliferation of the potentially harmful *S. aureus*. The results are summarized in Figure 4. This analysis helps us understand not only whether a product is an irritant but also how compatible it is with the health of the skin microbiome. This is particularly crucial for sensitive or infection-prone skin. In the conducted analyses, it was observed that adult diapers containing postbiotics maintained the balance in favor of *S. epidermidis*. Microbial balance studies confirmed that postbiotic formulations supported a favorable *S. epidermidis*/*S. aureus* ratio (e.g., *S. epidermidis* ≈ 3.5 × 10^2^ vs. *S. aureus* ≈ 2.4 × 10^1^ CFU/mL), demonstrating a clear predominance of *S. epidermidis* in the postbiotic-treated samples.

#### 3.4.3. Microbial Diversity

**Figure 5:**
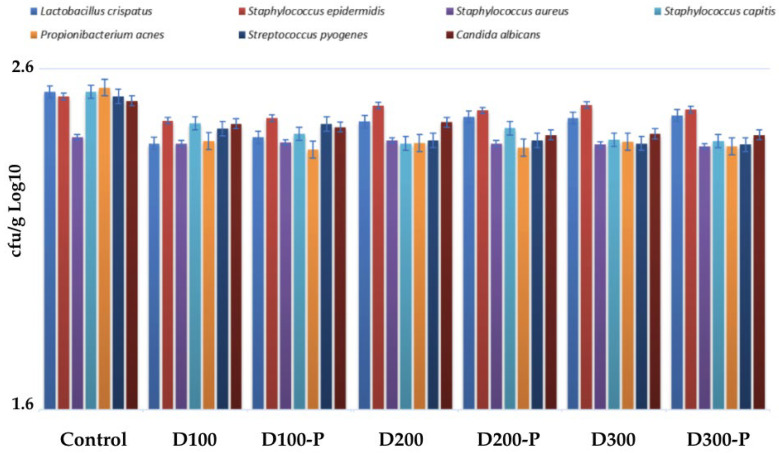
Graphical representation of Microbial Diversity Results of Groups.

Based on the analyses, a slight increase in the levels of some potentially harmful microorganisms (especially *S. aureus*) was observed in the postbiotic-free samples (e.g., D100). These diapers may have the potential to slightly disrupt the skin’s natural microbiome balance. In the postbiotic-containing samples (e.g., D100-P), the levels of beneficial bacteria like *S. epidermidis* were maintained, while the levels of potentially harmful microorganisms such as *S. aureus* and *C. albicans* were observed to be either stable or slightly reduced compared to the control group. The results are summarized in Figure 5. This indicates that postbiotics support a healthy balance of the skin microbiome. In addition postbiotic formulations preserved microbial diversity (*Lactobacillus*, *Cutibacterium*, *Streptococcus*, *Candida* species). No significant loss of microbiota richness compared to controls was observed.

In the overall evaluation, postbiotic-embedded samples showed stable incorporation of bioactive compounds (OPU & lactic acid) non-cytotoxic and non-irritant classification, and positive modulation of microbiota balance. Collectively, ATA-coded postbiotic diaper prototypes can be classified as “microbiota-friendly and skin-safe” products.

## 4. Discussion

Our gravimetric OPU determinations (0.64–0.68%) and HPLC-UV analyses of lactic acid (0.0041–0.0045%) confirmed the reproducible incorporation of postbiotic compounds into nonwoven substrates. The narrow variability between replicates (RSD < 1%) and the absence of inter-analyst differences suggest that the embedding process is both stable and scalable, supporting the feasibility of developing hygiene products with controlled postbiotic delivery. Comparable research has demonstrated that various biological agents can be incorporated into textile materials [46,47]. In contrast, our work represents the first demonstration of stable incorporation of postbiotics into nonwoven hygiene substrates.

In addition to stability, biocompatibility was further evaluated to assess potential cytotoxic effects. Both keratinocyte cytotoxicity assays (ISO 10993-5) and reconstructed human epidermis irritation assays (OECD 439) showed that postbiotic-containing samples were non-cytotoxic and non-irritant, with cell viability consistently ≥95–100%, markedly higher than the positive controls (SDS ≤ 10%). These results indicate that adding postbiotics not only prevents toxicity but may also enhance cell survival compared to non-postbiotic controls (~65–70%). The safety and cytotoxic effects of postbiotics on keratinocytes have also been examined in recent studies, which generally reported minimal cytotoxicity and even highlighted anti-inflammatory and barrier-protective properties [48,49].

Having established stability and safety, microbiological interaction studies were conducted to investigate effects on skin commensals and pathogens. Postbiotic-containing prototypes preserved microbial diversity, showing no adverse impact on representative skin species (*L. crispatus*, *P. acnes*, *S. epidermidis*, *C. albicans*). Notably, postbiotic formulations promoted a favorable equilibrium between *S. epidermidis* and *S. aureus*, with commensal *S. epidermidis* populations being maintained. Such selective modulation aligns with the concept of “skin microbiota-friendly” products, which support commensals while limiting opportunists. Our findings are consistent with recent microbiological studies, which demonstrate that *S. epidermidis* can suppress *S. aureus* colonization and pathogenicity through competitive exclusion and immune modulation, underscoring the importance of this balance for maintaining cutaneous integrity [50].

Earlier research has shown that *Lactobacillaceae*-derived postbiotics in topical formulations strengthen the epithelial barrier, increase tight junction protein expression, and mitigate inflammation [51]. These findings are consistent with the barrier-protective effects observed in our textile-based prototypes. Similarly, Martorell et al. (2021) investigated that heat-inactivated *Bifidobacterium longum* postbiotics retained functional properties and provided protection against oxidative stress and inflammation [52]. However, stability issues were reported in some formulations. In addition, da Silva Vale et al. (2023) demonstrated that cream formulations containing postbiotics provided barrier support, wound healing, and anti-aging benefits [53]. Duarte et al. (2022) reviewed the antioxidant and anti-inflammatory properties of these compounds in topical applications [54].

Building on these contributions, our results demonstrate for the first time that postbiotics can be safely and stably integrated into nonwoven hygiene substrates, preserving both functional stability and biological activity.

Together, these findings highlight the broader implications of our work for personal hygiene and skin health. Postbiotics are known to suppress inflammatory markers, such as IL-1 and IL-6, support the structure of the stratum corneum through the action of short-chain fatty acids, and improve outcomes in inflammatory skin conditions, including atopic dermatitis, eczema, and acne [45,55,56,57]. While preserving the skin’s natural microbiome balance, postbiotics contain substances like bacteriocins and organic acids that help prevent the proliferation of potentially harmful pathogens. This can contribute to controlling bacteria like *Cutibacterium acnes*, which cause acne [58]. Some studies suggest that postbiotics have antioxidant properties and show potential for combating photoaging [59]. This also demonstrates that the health benefits of postbiotics are pretty extensive, ranging from food to agriculture, cosmetics to skin health [60]. The extensive advantages of postbiotics, already explored in food, agriculture, and cosmetics, are here extended to hygiene textiles. By integrating postbiotics into adult diapers, we demonstrate a dual advantage: the functional stability of bioactive compounds within the fabric and tangible health benefits through preservation of microbiota balance and skin compatibility. Given the vulnerability of adult diaper users to dermatitis and dysbiosis, postbiotic-containing prototypes may offer a clinically relevant innovation. Future *in vivo* studies will be essential to validate these *in vitro* findings and to confirm their long-term dermatological and geriatric benefits.

## 5. Conclusions

This study demonstrated that postbiotic incorporation into nonwoven fabrics and adult diaper prototypes is technically feasible, chemically stable, and biologically safe. Gravimetric OPU and HPLC-UV analyses confirmed consistent embedding of postbiotic metabolites, particularly lactic acid, with reproducible results across replicates and analysts.

*In vitro* cytotoxicity and epidermal irritation assays revealed that postbiotic-containing samples maintained ≥95–100% cell and tissue viability, classifying them as non-cytotoxic and non-irritant. Furthermore, microbiota interaction studies highlighted that these formulations preserved microbial diversity and supported a favorable balance between commensal *S. epidermidis* and pathogenic *S. aureus.*

Collectively, ATA-coded postbiotic-embedded adult diaper prototypes can be considered microbiota-friendly, skin-compatible, and functionally innovative hygiene products. These findings support the potential of postbiotics as health-promoting agents in personal care applications. Future *in vivo* and clinical studies are recommended to confirm their efficacy in reducing skin irritation and supporting microbiota homeostasis in diaper users.

## Figures and Tables

**Table 1:** Microbial safety assessment results for diaper samples.

Analysis	Result (CFU/g)	Limit for Products Used Around the Eyes and for Children Under 3 Years	Limit for Other Products	Compliance Status
Total Aerobic Mesophilic Bacterial Count	<1.0 × 10^1^	1.0 × 10^2^	1.0 × 10^3^	Complies
*Pseudomonas aeruginosa* Detection	Not detected	Absent	Absent	Complies
*Escherichia coli* Detection	Not detected	Absent	Absent	Complies
*Candida albicans* Detection	Not detected	Absent	Absent	Complies
*Staphylococcus aureus* Detection	Not detected	Absent	Absent	Complies
Total Yeast and Mold Count	<1.0 × 10^1^	1.0 × 10^2^	1.0 × 10^3^	Complies

## Data Availability

The original contributions presented in this study are included in the article. Further inquiries can be directed to the corresponding author.

## Appendix A

**Table A1:** Gravimetric Determination of OPU Levels and Reproducibility.

MATRIX	NON WOVEN FABRIC (2.2 mL)		NON WOVEN FABRIC (2 mL)	
OPU%	Analyst-1	Analyst-2	Analyst-1	Analyst-2
	0.673	0.666	0.645	0.655
	0.680	0.669	0.642	0.646
	0.667	0.680	0.648	0.641
	0.667	0.667	0.639	0.644
	0.676	0.676	0.641	0.646
	0.673	0.667	0.651	0.642
MEAN	0.673	0.671	0.644	0.645
STANDARD DEVIATION	0.005	0.006	0.005	0.005
MIN	0.667	0.666	0.639	0.641
MAX	0.680	0.680	0.651	0.655
GRUBBS LOW (G)	1.159	0.788	1.202	0.918
GRUBBS HIGH (G)	1.427	1.581	1.421	1.857
GRUBBS CRITERION (GÖ)	1.890	1.890	1.890	1.890
EVALUATION (G<GÖ)	TRUE	TRUE	TRUE	TRUE
RSD	0.008	0.008	0.007	0.008
% RSD	0.750	0.848	0.712	0.775
Decimal Mean	0.007	0.007	0.006	0.006
PRSD	4.246	4.247	4.273	4.272
1/2PRSD	2.123	2.124	2.137	2.136
EVALUATION (%RSD<1/2PRSD)	TRUE	TRUE	TRUE	TRUE

**Table A2:** Determination of Lactic Acid.

MATRIX	NON WOVEN FABRIC (2.2 mL)		NON WOVEN FABRIC (2 mL)	
LACTIC ACID %	Analyst-1	Analyst-2	Analyst-1	Analyst-2
	0.0044	0.0045	0.0042	0.0040
	0.0045	0.0042	0.0041	0.0041
	0.0044	0.0044	0.0042	0.0040
	0.0043	0.0045	0.0041	0.0041
	0.0043	0.0044	0.0040	0.0042
	0.0045	0.0045	0.0041	0.0041
MEAN	0.0044	0.0044	0.0041	0.0041
STANDARD DEVIATION	0.0001	0.0001	0.0001	0.0001
MIN	0.0043	0.0042	0.0040	0.0040
MAX	0.0045	0.0045	0.0042	0.0042
GRUBBS LOW (G)	1.2898	1.7699	1.0349	1.0747
GRUBBS HIGH G)	1.2263	0.8337	1.2571	1.5503
GRUBBS CRITERION (GÖ)	1.8900	1.8900	1.8900	1.8900
EVALUATION (G<GÖ)	TRUE	TRUE	TRUE	TRUE
RSD	0.0236	0.0238	0.0197	0.0166
% RSD	2.3555	2.3841	1.9671	1.6608
Decimal Mean	0.0000	0.0000	0.0000	0.0000
PRSD	9.0497	9.0483	9.1373	9.1545
1/2PRSD	4.5248	4.5241	4.5686	4.5773
EVALUATION (%RSD<1/2PRSD)	TRUE	TRUE	TRUE	TRUE
